# Temozolomide-induced Aplastic Anemia Treated with Eltrombopag and Granulocyte Colony Stimulating Factor: A Report of a Rare Complication

**DOI:** 10.7759/cureus.3329

**Published:** 2018-09-18

**Authors:** Shauna L Newton, Kadra Kalamaha, Hermina D Fernandes

**Affiliations:** 1 Internal Medicine, University of North Dakota School of Medicine and Health Sciences, Bismarck, USA

**Keywords:** temozolomide, aplastic anaemia, glioblastoma multiforme (gbm), eltrombopag, stupp protocol

## Abstract

Temozolomide is an alkylating agent used in the treatment for glioblastoma multiforme (GBM), the most frequent primary malignant brain tumor in adults. Temozolomide was approved in March 2005 for treatment of GBM, with the Stupp protocol (radiotherapy and concomitant use of temozolomide). Despite initial studies demonstrating mild and well-tolerated side effects, several recent reports describe severe hematologic adverse effects associated with temozolomide use. We report the case of a 51-year-old female diagnosed with GBM who received the standard treatment protocol of radiotherapy and concomitant temozolomide. The patient developed prolonged pancytopenia. Bone marrow biopsy demonstrated hypocellular bone marrow with diminished trilineage hematopoiesis, suggestive of drug-induced aplastic anemia. Although temozolomide is regarded as a safe drug with few side effects, severe hematologic toxicities have been reported.

## Introduction

Temozolomide is an oral alkylating agent used as the first-line treatment for glioblastoma multiforme (GBM) [1]. Before its approval, standard therapy consisted of surgical resection, radiotherapy, and adjuvant carmustine [2]. The United States Food and Drug Administration (FDA) initially approved temozolomide in March 2005 [1]. The common side effects are well tolerated and include nausea, vomiting, constipation, fatigue, headache, with some of the more serious complications including myelosuppression and seizures [3]. Myelosuppression is the most severe side effect but considered dose limiting and reversible [1]. The Stupp protocol, which consists of radiotherapy with concomitant use of temozolomide followed by adjuvant temozolomide, is currently the standard of care [2]. Grade 3 and 4 hematologic toxic effects were observed in 14% of patients receiving adjuvant temozolomide therapy and in 7% of patients receiving concomitant radiotherapy with temozolomide during the clinical trial. No grade 3 or 4 hematologic toxic effects were observed with radiotherapy alone. Since its approval, several case studies have reported severe hematologic adverse events associated with temozolomide use. We describe the occurrence of aplastic anemia in a patient with GBM treated with surgical resection followed by concomitant temozolomide and radiation therapy.

## Case presentation

A 51-year-old female with a history of asthma, hypertension, dyslipidemia, and atherosclerotic heart disease presented to the emergency department with seizures. Through magnetic resonance imaging (MRI) of the brain, three left-sided intracranial lesions associated with mild vasogenic edema were discovered. Because of the metastatic appearance of lesions and her history of smoking, computed tomography (CT) imaging of the chest, abdomen, and pelvis was performed to evaluate for other sites of primary malignancy. However, CT imaging showed no other sites of a primary malignancy. The patient was placed on levetiracetam for seizure prophylaxis and dexamethasone for treatment of vasogenic edema. After an evaluation by neurology and neurosurgery, she underwent a left-sided craniotomy for removal of a left frontal brain lesion. Initial frozen sections were suspicious for primary brain malignant neoplasm, and final pathology confirmed the diagnosis of GBM (WHO grade IV).

Her initial treatment plan was temozolomide 75 mg/m^2^ once per day with concurrent radiation therapy, given in 30 fractions of 2 gray (Gy) five days per week for a total dose of 60 Gy, to be followed four weeks later by adjuvant temozolomide monotherapy at 150 mg/m^2^ a day for five days of a 28-day cycle [1,2]. Due to complications with insurance authorization, the temozolomide was started a week after her radiation therapy start date. Therefore, the patient received six weeks of radiation therapy and five weeks of temozolomide. No cytopenias were noted during her treatment course. She was prescribed trimethoprim-sulfamethoxazole (TMP-SMX) 800-160 mg once a day on Monday, Wednesday, and Friday for prophylaxis of Pneumocystis jiroveci infections. Home medications during concurrent temozolomide and radiation therapy included nebivolol, hydrocodone-acetaminophen, pantoprazole, triamterene-hydrochlorothiazide, lisinopril, albuterol, simvastatin, levetiracetam, dexamethasone, ondansetron, and TMP-SMX.

On day 54 following initial dose of temozolomide, she presented to the emergency department with epistaxis. Her complete blood cell count showed pancytopenia with a white blood cell count (WBC) of 0.4 K/µL (normal range: 4.0–11.0 K/µL), hemoglobin of 8.5 g/dL (normal range: 11.5–15.8 g/dL), and platelet count of 6 K/µL (normal range: 140–400 K/µL). The epistaxis was controlled with pressure and wound seal powder. The patient was also given two units of platelets for treatment of thrombocytopenia, which brought her platelet count to 78 K/µL (normal range: 140–400 K/µL). She was subsequently discharged home and instructed to follow up with oncology. During the follow-up appointment, her medication list was reviewed. The patient reported non-compliance with her oral TMP-SMX pill as prescribed. She was instructed to discontinue oral TMP-SMX and triamterene-hydrochlorothiazide to reduce the possibility of other drug-induced cytopenias.

On day 65 following initial dose of temozolomide, she continued to remain pancytopenic. She presented with confusion and altered mental status consistent with a postictal state and was hospitalized for concomitant hematemesis. She was febrile with a temperature of 101.7°F, pulse rate of 104 beats per minute, blood pressure of 93/73 mmHg, and respirations of 20 per minute. Abnormal lab values included a total WBC count of 0.7 K/µL (normal range: 4.0–11.0 K/µL), percentage of neutrophils at 1.5% with absolute neutrophil count (ANC) of 10.5 cells/µL, red blood cell count of 2.22 million/µL (normal range: 3.80–5.30 million/µL), hemoglobin of 7.1 g/dL (normal range: 11.5–15.8 g/dL), and platelet count of 11 K/µL (normal range: 140–400 K/µL) consistent with pancytopenia due to severe bone marrow suppression. The etiology of the pancytopenia was thought to be secondary to drug side effects and therefore, levetiracetam was discontinued and seizure prophylaxis was switched to lacosamide. Similar to aplastic anemia, the degree of bone marrow suppression was classified as “very severe” with depression of at least two of three cell lines, and ANC less than 200 cells/µL [4]. Since the pancytopenia persisted beyond 28 days after the last temozolomide dose (despite discontinuation of TMP-SMX, triamterene-hydrochlorothiazide and levetiracetam), it was attributed to temozolomide-induced bone marrow suppression [4]. Additional abnormalities include a chloride of 95 mEq/L (98–110 mEq/L), blood urea nitrogen of 28 mg/dL (normal range: 7–22 mg/dL), alanine amino transferase of 54 U/L (normal range: <40 U/L), albumin of 2.8 g/dL (normal range: 3.5–5.0 g/dL), C-reactive protein of 498 mg/L (normal range: <5.0 mg/L), glomerular filtration rate of 51 mL/min/1.73 m^2^ (normal range: >=60 mL/min/1.73 m^2^) and a prolactin 37.1 ng/mL (normal range: <23.0 ng/mL). Prothrombin time/International normalized ratio, troponin, ammonia, thyroid stimulating hormone, free T4, and partial thromboplastin time were within normal limits. Imaging studies with a CT brain to evaluate altered mentation in the setting of thrombocytopenia, showed no acute intracranial hemorrhage.

The patient was given supportive platelet transfusions to keep platelet counts greater than 50 K/µL, due to active clinical bleeding with hematemesis. She was given supportive erythropoietic transfusions with packed red blood cells (PRBCs). She was started on Granulocyte-Colony Stimulating Factor (G-CSF) to stimulate granulocytopoiesis and improve WBC counts. No further adjuvant temozolomide was administered. An esophagogastroduodenoscopy (EGD) was performed to evaluate etiology of hematemesis. This revealed esophageal ulcers with pathologic findings consistent with herpes simplex virus (HSV) esophagitis. Given her febrile neutropenia, she was started on empiric treatment with intravenous cefepime. She was also started on antifungal prophylaxis with fluconazole due to ongoing immunosuppression. The HSV esophagitis was treated with oral acyclovir. Her HSV esophagitis and fevers resolved with this treatment.

The patient continued to receive supportive treatment with G-CSF injections, PRBC, and platelet transfusions. A bone marrow biopsy was performed due to persisting pancytopenia on day 71 following initial dose of temozolomide. However, this was non-diagnostic due to dilute specimen. A repeat bone marrow biopsy was subsequently performed on day 77. The bone marrow biopsy and aspirate revealed predominantly trabecular bone with very little intact bone marrow. Myeloid maturation was markedly diminished and demonstrated a left shift. There were no increased blasts. Erythropoiesis was markedly diminished but without evidence of dyserythropoiesis. Rare nuclear irregularity was noted. Megakaryocytes were scarce to essentially absent. There were occasional lymphocytes and monocytes present. No significantly increased pathologic ringed sideroblasts were identified. Cellularity was estimated to be at less than 15%. Trilineage maturation was decreased overall. Normal female cytogenetics were noted. Flow cytometry revealed left shifted, immature granulocytes with CD56 expression on granulocytes and monocytes. These findings on her bone marrow biopsy revealed an aplastic or hypoplastic cause of pancytopenia, and were supportive of an acquired aplastic anemia.

After confirming the etiology of pancytopenia by bone marrow biopsy, the oral thrombopoietin agonist eltrombopag was started to aid in count recovery while formulating a treatment plan for consideration of immunosuppression with cyclosporine and anti-thymocyte globulin (ATG) [4,5]. In addition to the eltrombopag, G-CSF injections, PRBC, and platelet transfusions were continued. Her ANC started to improve three days following initiation of eltrombopag with resolution of neutropenia nine days following initiation. She continued to exhibit an improvement of anemia requiring infrequent PRBC transfusions, and protracted but improving thrombocytopenia. G-CSF support was continued daily until her ANC improved to greater than 1500 cells/µL and then discontinued. The eltrombopag was discontinued after four weeks of use due to transaminitis. She was discharged from hospital due to a reasonable degree of hematopoietic recovery at day 131 following first dose of temozolomide. At the time of discharge, she had a stable and moderate anemia without transfusion support, had recovered her neutrophil counts, and platelet counts had recovered to 30 K/µL requiring infrequent (one to two times per month) platelet transfusions for goal platelets greater than 20 K/µL. Therefore, consideration of allogeneic hematopoietic stem cell transplantation (alloHSCT) was deferred. On day 314 following first dose of temozolomide, her platelet counts continued to improve, and were in the range of 80–90 K/µL unsupported by platelet transfusions.

Bevacizumab was considered as second-line treatment for her GBM but was not started when platelet counts were under 50 K/µL because of the risk of fatal hemorrhage in the setting of thrombocytopenia. Bevacizumab is known to inhibit vascular endothelial growth factor (VEGF) resulting in decreased matrix deposition in blood vessels, making them susceptible to bleeding [6]. Maintenance therapy with tumor-treating fields (TTF) with temozolomide had now been approved for newly diagnosed glioblastomas after surgery [7]. Our patient was then started on antineoplastic therapy with TTF without temozolomide to treat her GBM to which she exhibited good disease control over a three-month time interval. She continued to exhibit hematopoietic recovery over this time interval with intermittent and infrequent platelet transfusions. At three months following TTF use, she exhibited disease progression of her GBM. Radiation or surgery was not option for treatment at the time, in the opinion of the treating surgical and radiation oncology teams, necessitating use of bevacizumab. At the time, her platelet counts were in the range of 80–90 K/µL unsupported by transfusions. One month following the start of bevacizumab, she developed infected wound flap at site of prior cranial surgery. She required a craniotomy for evacuation of subdural infection with drains. She was also placed on intravenous ceftriaxone and metronidazole for six weeks. Bevacizumab was deferred to allow for wound healing. Unfortunately, during this time interval she experienced acute respiratory failure needing hospitalization due to respiratory syncytial virus pneumonia. Due to declining quality of life from multiple medical issues, the patient and family elected to pursue hospice. She passed away 16 months from initial diagnosis of GBM due to disease progression from malignancy.

A complete summarization of the timeline of events has been presented in Table 1.

**Table 1:** Timeline of events. WBC: White blood cell; ANC: Absolute neutrophil count; RT: Radiation therapy; G-CSF: Granulocyte-colony stimulating factor; TTF: Tumor-treating fields; GBM: Glioblastoma multiforme.

Timeline	Day of treatment	WBC count (K/µL)	Hemoglobin (g/dL)	Platelet (K/µL)	ANC (cells/µL)
RT start	-7	-	-	-	-
Temozolomide start	1	12.6	14.2	300	9500
During concurrent temozolomide/RT	27	8.4	14.3	302	6400
Final week of temozolomide/RT	35	6.7	13.3	107	5300
Emergency room with epistaxis, nadir	54	0.4	8.5	6	0
Hospitalized, G-CSF injection	65	0.2	7.0	29	0
Bone marrow biopsy, non-diagnostic	71	0.2	8.6	6	0
Bone marrow biopsy, repeat	77	0.7	8.2	5	0
Eltrombopag	86	0.5	8.1	5	0
Neutrophil recovery	89	0.4	8.2	17	100
Resolution of neutropenia	95	2.0	7.6	16	1500
Hospital discharge	131	5.1	8.1	29	3300
TTF therapy	194	3.2	9.1	23	2500
GBM progression, bevacizumab	314	4.3	12.6	81	3100
Hospice	378	3.4	10.6	57	2600

## Discussion

GBM is the most frequent primary malignant brain tumor in adults [2]. The median survival of patients with GBM is generally one year from the time of diagnosis, with most patients succumbing to the illness within two years [2]. The clinical trial of the Stupp protocol showed a 37% relative reduction in the risk of death and a clinically meaningful median overall survival of 14.6 months for patients treated with radiotherapy plus temozolomide as compared with those who received radiotherapy alone [2]. The statistically significant increase in survival resulted in temozolomide approval for first-line treatment of GBM.

Temozolomide is an oral alkylating agent, which shares a cytotoxic mechanism with dacarbazine and other methylating compounds [8]. Its active metabolite, 5-(3-methyltriazen-1-yl) imidazole-4-carboxamide (MTIC), methylates the N^7^ and O^6^ position of guanine and the O^3^ position of adenine [9]. Methylation of the O^6^ position of guanine stimulates the activity of the base excision repair protein, methylguanine-DNA methyltransferase (MGMT). MGMT subsequently demethylates this position on guanine, resulting in inactivation of the enzyme [9]. Daily temozolomide therapy at a dose of 75 mg/m^2^ depletes MGMT levels, resulting in the activation of alternative repair pathways [2]. Mismatch repair enzymes attempt to repair the methylated DNA molecule, but failed attempts result in fragmentation of DNA and apoptosis of the cell [9]. Studies have demonstrated that lower levels of MGMT are associated with prolonged survival in patients with GBM [2]. One report observed that methylation of the MGMT promoter is associated with a substantial survival benefit in patients treated with concomitant temozolomide and radiotherapy [2]. Radiotherapy is known to induce MGMT [2]. Therefore, the addition of temozolomide allows the treatment modalities to work synergistically.

A similar mechanism is proposed to explain temozolomide-related bone marrow suppression [8]. Higher levels of MGMT are thought to protect cells from temozolomide cytotoxicity [8]. As stated above, when MGMT is present, it removes the methyl group from the O^6^ guanine position of DNA to repair the molecule. Bone marrow precursors have low levels of MGMT, which increases the susceptibility of these cells to temozolomide [8]. A study published by Sabharwal et al. noted that low levels of pretreatment MGMT in peripheral blood cells were associated with an increased risk of thrombocytopenia and neutropenia with temozolomide use [8]. Hematologic toxicity is a well-reported side effect of temozolomide, with thrombocytopenia, leukopenia, and neutropenia being most common [2,10]. Aplastic anemia has also been described [10]. However, the myelosuppression induced by temozolomide is thought to be dose limiting and reversible after two weeks of discontinuing the drug [11]. Overall, temozolomide is regarded as a safe and well-tolerated drug with the most serious side effects occurring in less than 5% of patients [1,3,10,12].

Aplastic anemia is a rare disease in which the hematopoietic stem cells of bone marrow are damaged. It results in pancytopenia (deficiency of erythrocytes, leukocytes, and platelets) [3]. Causes of aplastic anemia can be inherited or acquired. Common causes of acquired aplastic anemia include infection, exposure to chemicals, and medications [3]. Some medications that are known to induce cytopenias include nonsteroidal anti-inflammatory drugs, sulfonamides, chloramphenicol, anti-thyroid drugs, furosemide, anti-convulsant drugs, corticosteroids, and allopurinol [3,13]. Destruction of hematopoietic precursors is thought to be immune-mediated, with drugs and other chemicals triggering an aberrant T-cell response targeting hematopoietic stem cells [11,14]. It is unclear whether this is driven by antigens or immunological disarray [14]. Additionally, predispositions to this immune response have been difficult to identify due to the rare nature of idiosyncratic drug reactions [14].

To diagnose aplastic anemia, both peripheral blood cytopenias and a bone marrow biopsy demonstrating hypocellular marrow must be present. The remaining bone marrow should be free of malignant infiltrates or evidence of megaloblastic hematopoiesis [15].

Villano et al. reported one of the first cases of temozolomide-induced aplastic anemia in 2006 [1]. The paper describes a 45-year-old male who underwent standard fractionated radiotherapy with concomitant temozolomide. After initiating temozolomide monotherapy, the patient developed profound pancytopenia [1]. A bone marrow biopsy revealed aplastic anemia. Causality of the aplastic anemia could not be definitively determined because the patient was also prescribed phenytoin and carbamazepine while undergoing treatment with temozolomide [1]. However, discontinuation of the drugs did not lead to a recovery of bone marrow suppression, which is uncommon with anticonvulsants. Prior to this case, one phase II trial documented a case of aplastic anemia with temozolomide treatment. The patient in this trial was concomitantly taking TMP-SMX with temozolomide, and therefore causality could not be determined with this case as well [1].

Since 2006, several additional case reports of temozolomide-induced aplastic anemia have been reported. In 2007, a drug safety letter was released by the FDA that reported 18 cases from 1999 to 2006 [3]. No patients in this newsletter had prior pancytopenia or aplastic anemia. One-third (six of eighteen) of these patients had been exposed to other drugs during treatment with temozolomide that may have contributed to the development of aplastic anemia. Only five patients experienced marrow recovery within one to four months of discontinuing temozolomide, and five other patients ultimately died from complications of aplastic anemia or complications related to allogeneic transplant [3]. A systematic review of temozolomide-related idiosyncratic and other uncommon toxicities was performed in 2012 [9]. This report analyzed 73 cases of temozolomide-induced toxicity. Of the 73 cases, 21 cases of hematologic idiosyncratic drug reactions were identified. Risk factors identified from this report include female gender and patients receiving temozolomide concomitantly with radiotherapy. Only three out of the 21 reported cases were not taking any medication known to have hematologic side effects [9]. In 2015, Mayo Clinic performed a cohort study with patients treated with temozolomide by Mayo Clinic Rochester from 2003 to 2014 plus 15 additional reported cases identified from the literature [4]. Of 2,356 patients treated at Mayo Clinic with temozolomide, only 15 patients (0.6%) developed bone marrow suppression. Therefore, 30 total patients were included in this study. The average age when bone marrow suppression was diagnosed was 55. Twenty-seven out of 30 patients (90%) received temozolomide with concomitant radiation, while only three single agent cases were found (10%). Consistent with the report from Dixit et al., the study also reported a female predominance (80%) [9]. Lastly, the study indicated that the majority of cases demonstrated temozolomide-induced bone marrow suppression after the first cycle of therapy (63%) [4].

Early diagnosis of temozolomide-induced aplastic anemia is imperative since it is associated with high morbidity and mortality [4]. Management begins with immediate discontinuation of causative agents [13]. Empiric broad-spectrum antibiotic therapy should be initiated to prevent sepsis or secondary infection [13]. Current treatment options include supportive therapy with transfusions and growth factors, eltrombopag, immunosuppressive therapy (IST) and alloHSCT [3-5,9,12]. Hematopoietic growth factors have been shown to shorten the duration of antibiotic therapy and drug count recovery. Frequently, IST with horse ATG and cyclosporine has been used to treat aplastic anemia, especially in those who are not eligible for alloHSCT [14]. Our patient described in the case report above recovered her neutrophil and erythrocyte counts with supportive transfusions, G-CSF injections and eltrombopag. Also, discontinuation of offending drugs has been known to play a role in the spontaneous recovery of drug-induced aplastic anemia, usually noted one to two months following discontinuation of drug [16]. Following the use of eltrombopag, her transfusion requirements were minimal with no infectious complications due to a normal neutrophil count, and therefore IST was deferred. The oral thrombopoietin agonist eltrombopag was initially developed to stimulate thrombopoiesis in patients with thrombocytopenia [4,5]. Through mouse models, the essential role of thrombopoietin in hematopoietic stem cell (HSC) hemostasis has been demonstrated [5]. It is theorized that eltrombopag acts not only to stimulate thrombopoiesis, but also to directly stimulate residual HSCs in patients with aplastic anemia [5]. In the cohort study by Kourelis et al., immunosuppressive treatment was attempted (two out of 30) without benefit, and patients on eltrombopag (one out of 30) demonstrated prolonged improvements in blood counts [4]. While alloHSCT may be curative, lethal complications related to transplant are not uncommon [1,4]. In younger patients who are refractory to treatment, alloHSCT is a potentially curative therapy [14].

## Conclusions

Temozolomide is an alkylating agent that is first-line therapy for the treatment of GBM. Although regarded as a relatively safe drug with a favorable side-effect profile, many cases of bone marrow suppression and aplastic anemia have been reported while using temozolomide. It is difficult to determine definitive causality in most of these cases because of the concomitant use of other medications known to induce aplastic anemia. However, some cases of aplastic anemia induced by temozolomide monotherapy have been described. The optimal treatment for temozolomide-induced aplastic anemia is not well defined due to its rare presentation. Physicians should be aware of this potentially fatal complication related to temozolomide and due consideration should be given when prescribing treatment for GBM. Patients should be monitored frequently with complete blood indices for early detection and treatment of this complication.
